# De Novo Splice Site Variant of TCF12 in a Boy With Isolated Kallmann Syndrome

**DOI:** 10.1155/crie/2350842

**Published:** 2025-07-03

**Authors:** Erina Suzuki, Hirohito Shima, Aki Ueda, Kazuhiko Nakabayashi, Keiko Matsubara, Yoko Kuroki, Junko Kanno, Maki Fukami

**Affiliations:** ^1^Department of Molecular Endocrinology, National Center for Child Health and Development, Tokyo, Japan; ^2^Department of Pediatrics, Tohoku University Graduate School of Medicine, Sendai, Japan; ^3^Division of Diversity Research, National Center for Child Health and Development, Tokyo, Japan; ^4^Department of Maternal-Fetal Biology, National Center for Child Health and Development, Tokyo, Japan; ^5^Department of Genome Medicine, National Center for Child Health and Development, Tokyo, Japan

**Keywords:** delayed puberty, gonadotropin deficiency, mutation, splice site

## Abstract

**Background:** Kallmann syndrome is a rare endocrinopathy characterized by congenital hypogonadotropic hypogonadism (CHH) and olfactory dysfunction. The current understanding of the genetic basis of Kallmann syndrome is fragmentary. TCF12 is a causative gene for autosomal dominant craniosynostosis with various complications. Although recent studies identified rare nucleotide substitutions and indels of TCF12 in a few families with CHH and additional clinical features, the significance of TCF12 variants as the cause of Kallmann syndrome remains uncertain.

**Case Presentation:** A Japanese boy exhibited bilateral cryptorchidism and micropenis at birth. He was otherwise healthy and had normal developmental milestones. At 11 years of age, he showed no pubertal signs. Physical examinations detected no clinical abnormalities except hyposmia. Brain imaging suggested olfactory bulb hypoplasia, but no other anomalies. A gonadotropin-releasing hormone (GnRH) stimulation test yielded low responses of gonadotropins. Whole-exome sequencing (WES) identified a hitherto unreported de novo heterozygous substitution at a splice acceptor site of TCF12 (c.391–1G >A). This variant was predicted to cause a frameshift and resultant premature termination (p.Ser132ProfsTer38) and was assessed as pathogenic, according to the ACMG/AMP 2015 guidelines. The patient carried no rare variants in other genes previously associated with Kallmann syndrome or CHH.

**Conclusion:** These results broaden the mutation spectrum of TCF12. More importantly, this study argues for the etiological relationship between TCF12 variants and isolated Kallmann syndrome.

## 1. Introduction

Kallmann syndrome is a rare endocrine disorder characterized by congenital hypogonadotropic hypogonadism (CHH) and olfactory dysfunction [1]. Kallmann syndrome results from monoallelic, biallelic, or oligogenic mutations in genes involved in hormone secretion from the hypothalamus or pituitary [1]. To date, more than 30 genes have been implicated in Kallmann syndrome or normosmic CHH, although pathogenic variants in these genes can explain less than half of the cases [1].

*TCF12* (NM_207037.2, alias *HTF4*) is a ubiquitously expressed gene that encodes a member of the basic helix-loop-helix (bHLH) transcription factor. *TCF12* is known as a causative gene for autosomal dominant craniosynostosis [2]. Previous studies have suggested that TCF12 heterodimerizes with TWIST1 and regulates specification of the boundary between neural crest and cephalic mesoderm [2]. Patients with heterozygous *TCF12* variants frequently exhibit skeletal malformations, facial dysmorphisms, and intellectual problems, in addition to craniosynostosis [3]. Some of these patients were reported to have CHH [3]. In 2020, Davis et al. [4] identified rare nucleotide substitutions and indels of *TCF12* in 13 families with CHH. Most variant-positive individuals exhibited Kallmann syndrome with dental, neurobehavioral, or musculoskeletal abnormalities. Subsequently, Celik et al. [5] have detected intragenic deletions of *TCF12* in a patient with Kallmann syndrome and thyroid tumor. These findings imply that *TCF12* is a candidate gene for Kallmann syndrome. However, there are no further reports of *TCF12* variants in patients with Kallmann syndrome. Since most patients reported by Davis et al. and Celik et al. [4, 5] exhibited complex phenotypes, the etiological relationship between *TCF12* variants and isolated Kallmann syndrome remains unknown. Here, we report a boy with a novel de novo splice site variant of *TCF12* and isolated Kallmann syndrome.

## 2. Case Presentation

The case was a Japanese boy born to a healthy, unrelated couple. At birth, he was noted to have bilateral cryptorchidism and micropenis. Endocrine evaluation at 1 month of age during minipuberty showed low levels of luteinizing hormone (LH), follicle-stimulating hormone (FSH), and testosterone (Table 1). Thus, he was suspected of having CHH and received testosterone injections and orchidopexy during infancy. He was otherwise healthy and showed normal development and growth. At 11 years and 4 months of age, he visited our hospital for clinical evaluation. His height and weight were 141 cm (−0.47 SD) and 45.4 kg (+0.90 SD). He could detect strong odors, but did not recognize any odors of foods or other items in daily life. He had no visual or hearing impairment. Physical examinations detected no signs of craniofacial, neurobehavioral, or musculoskeletal abnormalities. He showed prepubertal external genitalia of Tanner stage I. Brain imaging suggested hypoplasia of the olfactory bulbs, but no other anomalies. He did not undergo further imaging examinations. Endocrine examinations revealed low gonadotropin responses to gonadotropin-releasing hormone (GnRH) stimulation, together with normal blood levels of other hormones examined (Table 1). He was clinically diagnosed with isolated Kallmann syndrome.

**Figure 1:**
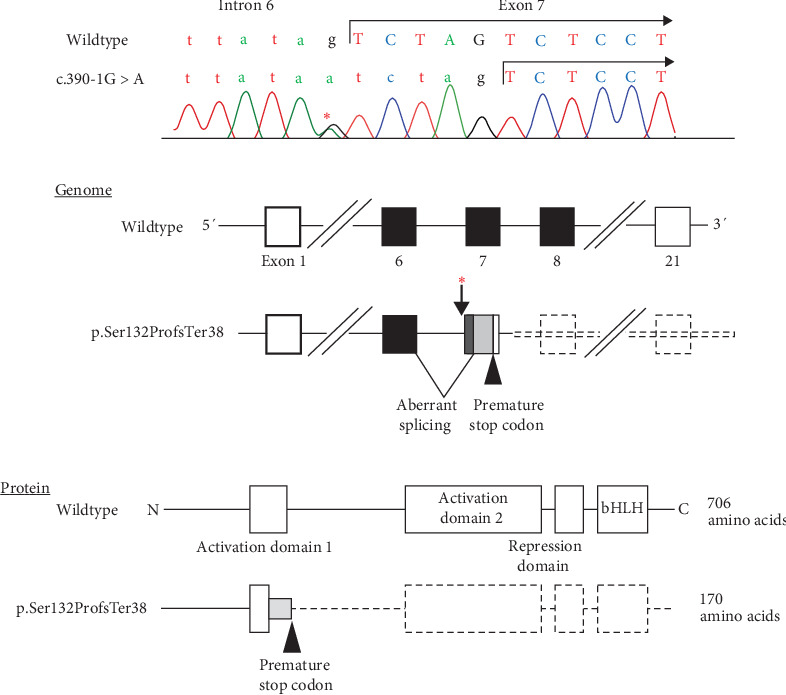
The *TCF12* variant identified in the patient. (A) Chromatogram of the variant. The c.390 -1G>A variant affected a consensus nucleotide at the splice acceptor site of exon 7. (B) The variant (indicated by the asterisk) was predicted to activate a nearby cryptic acceptor site leading to frameshift and premature termination (p.Ser132ProfsTer38). The black and white boxes indicate translated and untranslated regions, respectively. The striped and gray boxes depict intronized and frameshifting nucleotides, respectively. It remains unknown whether exons 8–21 of the mutant allele constitute an untranslated region. The exons and introns are not drawn to scale. (C) Predicted protein structure. The wildtype TCF12 protein contains two activating domains, one repression domain, and the basic helix-loop-helix (bHLH) domain. The variant was predicted to undergo nonsense-mediated mRNA decay, and even if the mRNA escapes decay, it would encode a truncating protein lacking the activation domain 2, the repression domain, and the bHLH domain. The gray box indicates aberrant amino acids. The domains are not drawn to scale.

The patient was identified through mutation screening of *TCF12* for patients with etiology-unknown CHH. We performed whole-exome sequencing (WES) for eight patients with Kallmann syndrome or normosmic CHH who carried no mutations in 14 major causative genes for CHH (*ANOS1*, *CHD7*, *FGF8*, *FGFR1*, *GNRH1*, *GNRHR*, *KISS1*, *KISS1R*, *PROK2*, *PROKR2*, *SOX10*, *TAC3*, *TACR3*, and *WDR11*). We searched for rare protein-altering variants in 57 genes reported as causative/candidate genes for CHH or Kallmann syndrome (Table S1) [6, 7]. The methods of WES and variant calling were reported previously [8]. As a result, we identified a rare *TCF12* variant in one patient (the present case). The patient carried a heterozygous c.391–1G > A variant, which affected a consensus nucleotide at a splice acceptor site of exon 7. The presence of the variant was confirmed by Sanger sequencing (Figure 1A). In silico prediction using SpliceAI (https://spliceailookup.broadinstitute.org/) suggested that the variant likely decreased the activity of the original splice site and instead activated a nearby cryptic splice acceptor site in exon 7. Hence, this variant was assumed to intronize the first five nucleotides of exon 7 to cause a frameshift from the 132nd codon and resultant premature termination at the 170th codon (p.Ser132ProfsTer38) (Figure 1B). The variant was not found in the Genome Aggregation Database (gnomAD, https://gnomad.broadinstitute.org/) and Tohoku Medical Megabank Organization Database (ToMMo, https://www.megabank.tohoku.ac.jp/). The variant was not identified in the parents of the patient. The variant was assessed as pathogenic (PVS1 + PM2 + PS2) according to the ACMG/AMP 2015 guidelines [9]. WES identified no rare variants in the remaining 56 genes.

## 3. Discussion

We identified a hitherto unreported de novo *TCF12* variant in a boy with Kallmann syndrome. The variant was assumed to activate a cryptic acceptor site in exon 7, leading to the intronization of the first five nucleotides of this exon. The mutant mRNA satisfies the condition of nonsense-mediated mRNA decay [10], and therefore, is likely to undergo rapid degradation. Even if the variant mRNA escapes nonsense-mediated decay, it would encode a truncating protein lacking the activation domain 2, the repression domain, and the bHLH domain (Figure 1C). Thus, this variant is likely to result in *TCF12* haploinsufficiency. These results broaden the mutation spectrum of *TCF12*.

The patient exhibited typical clinical features of Kallmann syndrome but lacked other craniofacial, neurobehavioral, or musculoskeletal abnormalities. Thus far, pathogenic *TCF12* variants have been identified mostly in patients with craniosynostosis or other developmental defects [2, 3]. Only two patients with frameshift or nonsense variants in *TCF12* (individuals 4 and 7 in [4]) were reported to have Kallmann syndrome without other complications. The present study provides evidence that heterozygous loss-of-function variants of *TCF12* are causes of isolated Kallmann syndrome. Since our patient carried no pathogenic variants in other known CHH-causative genes, monoallelic *TCF12* variants appear to be sufficient to cause Kallmann syndrome as a Mendelian disorder. Consistent with this, animal studies have shown that *Tcf12* is abundantly expressed in the embryonic ectoderm and neural folds, and loss of *Tcf12* perturbs GnRH neuronal patterning in zebrafish [4]. Since TCF12 is a transcription factor [2], it may transactivate some genes involved in the development of GnRH neurons. In this context, Davies et al. [4] have proposed that TCF12 interacts with other GnRH neuron-associated molecules such as STUB1 and SOX10. The underlying mechanisms of Kallmann syndrome in *TCF12*-mutation-positive patients need to be clarified in future studies.

Two matters are noteworthy for the *TCF12* variant in the present case. First, the patient was identified through WES for eight patients with etiology-unknown CHH. These results, in conjunction with previous findings that only 13 of 729 pedigrees with Kallmann syndrome carried *TCF12* variants [4], indicate that *TCF12* abnormalities account for only a small percentage of the etiology of Kallmann syndrome. Second, the patient lacked craniosynostosis or other malformations despite having a protein-truncating variant in *TCF12*. Thus far, no apparent genotype–phenotype correlation has been observed in *TCF12* abnormalities [4]. Future studies are needed to clarify the phenotypic determinants of this condition.

We identified a novel de novo splice site variant of *TCF12* in a boy with Kallmann syndrome without other clinical features. Our data broaden the mutation spectrum of *TCF12*. More importantly, this study argues for the etiological relationship between *TCF12* variants and isolated Kallmann syndrome.

## Figures and Tables

**Table 1:** Endocrine data of the patient.

Hormone	Patient		Reference values	
	Baseline	Peak^a^	Baseline	Peak^a^
One month of age				
LH (IU/L)	**<0.10**	—	1.67–4.23^b^	—
FSH (IU/L)	**0.37**	—	0.93–2.03^b^	—
Testosterone (nmol/L)	**<0.1**	—	2.77–11.8^b^	—
11.5 years of age				
LH (IU/L)	0.1	0.7	<0.1–0.4^c^	0.4–6.0^c^
FSH (IU/L)	**<0.10**	**3.3**	0.6–3.0^c^	6.3–15.6^c^
12 years of age				
TSH (mlU/L)	**4.01**	—	0.45–4.20^b^	—
IGF-1 (ng/mL)	**226**	—	113–579^b^	—

*Note*: Hormone values below the reference range are boldfaced.

Abbreviations: FSH, follicle-stimulating hormone; IGF-1, insulin-like growth factor 1; LH, luteinizing hormone; TSH, thyroid-stimulating hormone.

^a^GnRH stimulation test (100 μg/m^2^, bolus i.v.; blood sampling at 0, 30, 60, 90, and 120 min).

^b^Reference values of age-matched boys.

^c^Reference values of prepubertal boys.

## Data Availability

The data that support the findings of this study are available upon request from the corresponding author (Maki Fukami). The data are not publicly available due to privacy or ethical restrictions.

## Supporting Information

Supporting Information Supporting Information Table S1. Known causative/candidate genes for congenital hypogonadotropic hypogonadism and Kallmann syndrome.
